# Prostaglandin receptor EP3 regulates cell proliferation and migration with impact on survival of endometrial cancer patients

**DOI:** 10.18632/oncotarget.23140

**Published:** 2017-12-09

**Authors:** Junyan Zhu, Fabian Trillsch, Doris Mayr, Christina Kuhn, Martina Rahmeh, Simone Hofmann, Marianne Vogel, Sven Mahner, Udo Jeschke, Viktoria von Schönfeldt

**Affiliations:** ^1^ Department of Obstetrics and Gynecology, University Hospital, LMU Munich, Munich, Germany; ^2^ Department of Gynecology and Obstetrics, Shanghai Jiao Tong University, School of Medicine, Renji Hospital, Shanghai, China; ^3^ Department of Pathology, University Hospital, LMU Munich, Munich, Germany; ^4^ Division of Gynecological Endocrinology and Reproductive Medicine, Department of Gynecology and Obstetrics, University Hospital, LMU Munich, Munich, Germany

**Keywords:** prostaglandin receptor EP3, endometrial cancer, prognosis, estrogen receptor β, Ras

## Abstract

**Background:**

Prostaglandin E2 (PGE2) receptor 3 (EP3) regulates tumor cell proliferation, migration, and invasion in numerous cancers. The role of EP3 as a prognostic biomarker in endometrial cancer remains unclear. The primary aim of this study was to analyze the prognostic significance of EP3 expression in endometrial cancer.

**Methods:**

We analyzed the EP3 expression of 140 endometrial carcinoma patients by immunohistochemistry. RL95-2 endometrial cancer cell line was chosen from four endometrial cancer cell lines (RL95-2, Ishikawa, HEC-1-A, and HEC-1-B) according to EP3 expression level. Treated with PGE2 and EP3 antagonist, RL95-2 cells were investigated by MTT, BrdU, and wound healing assay for functional assessment of EP3.

**Results:**

EP3 staining differed significantly according to WHO tumor grading in both whole cohort (p = 0.01) and the subgroup of endometrioid carcinoma (p = 0.01). Patients with high EP3 expression in their respective tumors had impaired progression-free survival as well as overall survival in both cohorts above. EP3 expression in the overall cohort was identified as an independent prognostic marker for progression-free survival (HR 1.014, 95%CI 1.003-1.024, p = 0.01) when adjusted for age, stage, grading, and recurrence. Treatment with EP3 antagonists induced upregulation of estrogen receptor β and decreased activity of Ras and led to attenuated proliferation and migration of RL95-2 cells.

**Conclusions:**

EP3 seems to play a crucial role in endometrial cancer progression. In the context of limited systemic treatment options for endometrial cancer, this explorative analysis identifies EP3 as a potential target for diagnostic workup and therapy.

## INTRODUCTION

With about 320,000 new cancer cases in 2012, endometrial cancer (EC) becomes the fifth most common tumor, following breast, colorectum, cervix uteri, and lung cancer. It represents 4.8% of cancer in women worldwide and is the most frequent gynecological carcinoma in developed regions [1]. Moreover, the incidence rate in USA is expected to increase from 19.1 per 10,000 in 2012 to 42.13 per 10,000 in 2030 [1, 2].

Obesity, nulliparity, late menopause, diabetes, and use of tamoxifen are the best-known risk factors of EC, which can be summarized into unopposed endogenous and exogenous estrogen [3]. Several prospective studies focusing on postmenopausal EC patients and healthy control women have demonstrated a notable positive correlation between circulation estradiol level and EC [4, 5]. Estrogen receptors (ER), mediating the effect of estrogen, play a key role in differentiation and invasion of EC [6].

In numerous cancers, chronic inflammation has been linked to tumor progression and was recently demonstrated for EC as well [7]. Risk reductions of EC have been associated with a high-frequency use of aspirin, a non-steroidal anti-inflammatory drug (NSAID), decreasing prostaglandin (PG) synthesis via inhibiting the activity of cyclooxygenases (COXs) [8], especially in obese women according to the latest meta-analysis [9]. COX2 mRNA, protein expression and prostaglandin E2 (PGE2) synthesis are notably elevated in EC compared to healthy endometrium [10, 11]. Moreover, PGE2 has been shown to promote proliferation and invasion in EC [12]. PGE2 exerts its biological actions via binding to its seven-transmembrane, G-protein coupled receptors (GPCRs), termed EP1, EP2, EP3, and EP4 [13]. EP3 is reported to regulate the cancerogenesis and progression in various cancer cells, such as human prostate [14], breast [15], liver [16], colon [17], oral cancer cells [18]. Although the uterus is one of the organs with most abundant EP3 [19], only little is known about the contribution of EP3 in EC so far [12].

The present study aimed to examine the EP3 expression in tissue samples of EC patients and its association with clinicopathologic characteristics and survival. Also, we tried to find the mechanism of EP3’s effect on EC using human EC cells and establish the rationale of PGE2’s tumor-promoting action in EC.

## RESULTS

### Patients characteristics

Detailed medical records of 140 EC patients including age, stage of disease, histology, and grading are listed in Table 1. The median follow-up was 82.71 months and during the follow-up period, 18 (12.9%) patients recurred and 36 (25.7%) died.

**Table 1:** Clinical characteristics of included patients (n=140)

Clinical characteristics	All patients (n=140) No. (%)
Age (Median) [years]	65.7
Follow up (Median) [months]	82.7
Histology	
Endometrioid	102 (72.9)
Serous	11 (7.9)
Mucinous	6 (4.3)
Mixed cell	19 (13.6)
Undifferentiated	2 (1.4)
FIGO stage	
I	104 (74.3)
II	9 (6.4)
III	23 (16.4)
IV	4 (2.9)
WHO grading	
1	67 (47.9)
2	46 (32.9)
3	27 (19.3)
Lymph node involvement	
No	119 (85.0)
Yes	16 (11.4)
Unknown	5 (3.6)
Metastasis at first dignosis	
No	117 (83.6)
Yes	11 (7.9)
Unknown	12 (8.6)
Recurrence	
No	110 (78.6)
Yes	18 (12.9)
Unknown	12 (8.6)

FIGO: International Federation of Gynecology and Obstetrics; WHO: World Health Organization.

### EP3 expression in EC and correlation with clinicopathological characteristics

**Figure 1:**
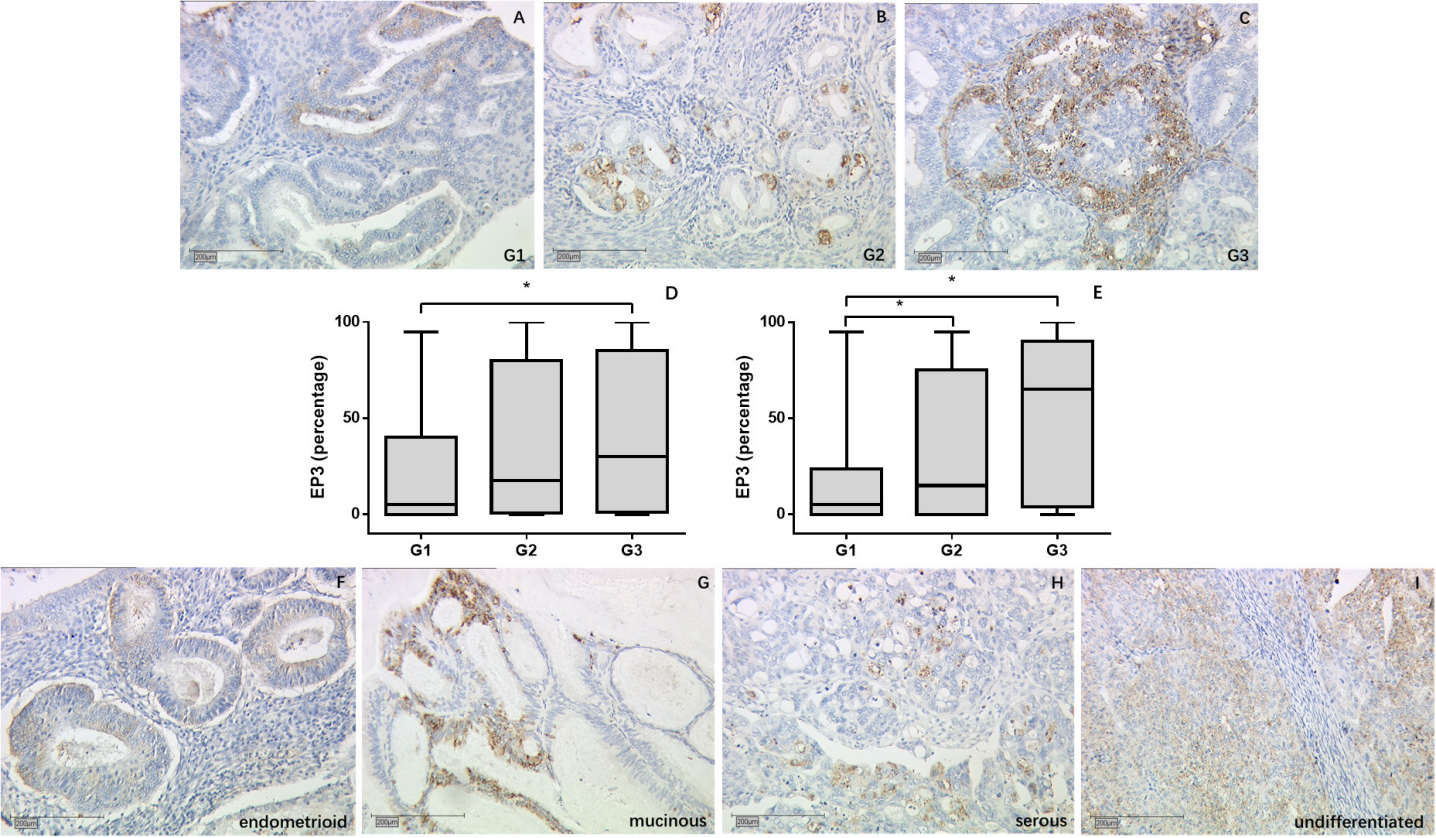
EP3 expression in EC tumor samples detected by immunohistochemistry **(A-C)** Representative microphotographs of EP3 staining in different grading of EC tissue. **(D, E)** EP3 expression is associated with grading with weakest staining in G1 tumors in overall cohort and endometrioid tumor subgroup. ^*^p < 0.05. **(F-I)** Representative microphotographs of EP3 staining in different histological subtypes of EC tissue. Scale bars equal 200 μm.

EP3 staining showed significant difference within the World Health Organization (WHO) grading in the overall cohort (p = 0.011) (Figure 1A-1D) as well as in the endometrioid adenocarcinoma subgroup (p = 0.013) (Figure 1E). In the overall cohort, the highest expression was in G3 (median = 30%), while the lowest expression was in G1 (median = 5%, p = 0.013). G2 staining showed no statistical differences compared to either G1 or G3 staining. The expression in endometrioid adenocarcinoma group followed the same trend. G1 staining (median = 5%) was much weaker than G2 (median = 15%, p = 0.041) and G3 staining (median = 65%, p = 0.013) and no differences were found between G2 and G3 group. EP3 expression among the different histological subtypes exhibited decreasing density for from undifferentiated (median = 45.5%), over mucinous cancer (median = 45%), serous carcinoma (median = 30%), mixed cell (median = 10%), and endometrioid histology (median = 7.5%), although the differences were not significant (Figure 1F-1I).

Besides that, no significant differences in EP3 expression were noted between different International Federation of Gynecology and Obstetrics (FIGO) stages. The expression of EP3 comparing cases being negative vs. positive for lymph node involvement or relapse was also not significantly different.

### Prognostic significance of EP3 in EC

**Figure 2:**
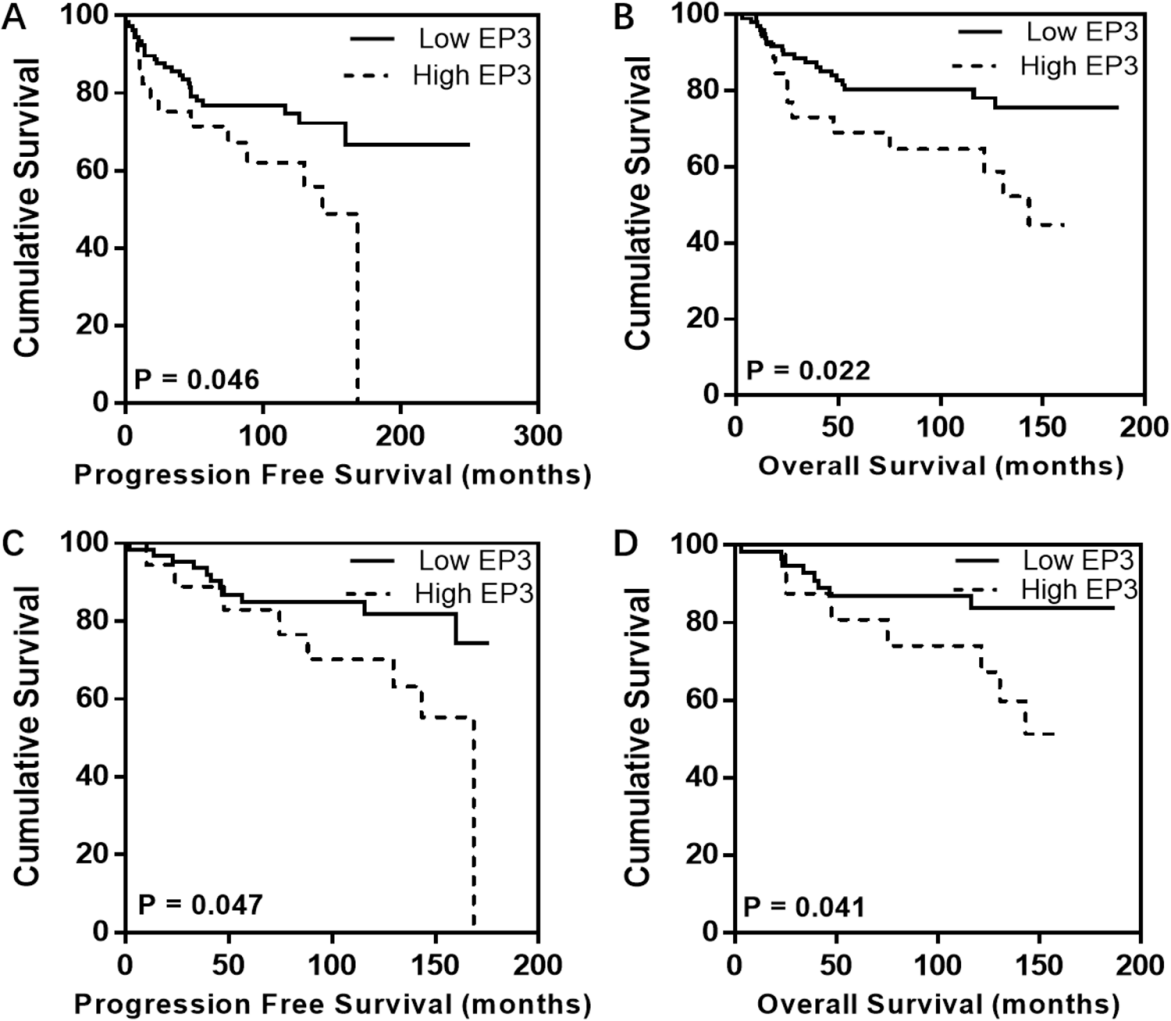
Kaplan-Meier analysis for EP3 in EC patients Individuals with high EP3 expression exhibit impaired PFS and OS in both overall cohort **(A, B)** and FIGO I endometrioid cancer group **(C, D)** compared to those with low EP3 expression.

A cut-off value of 72.5 was identified by using Receiver Operating Characteristic (ROC) curve. The staining percentage below 72.5 was defined as the low EP3 expression in 111 EC tissues (79.3%), while the one above 72.5 as the high EP3 expression was identified in 29 EC tissues (20.7%). Survival analysis was performed in the whole cohort as well as in specific subgroups such as FIGO I, endometrioid cancers, and FIGO I endometrioid cancers groups. Kaplan-Meier analysis indicated that patients with high expression in tumor had impaired progression-free survival (PFS) and overall survival (OS) in the overall cohort (10-year-PFS rate, 62.1% vs. 74.7%, p=0.046, Figure 2A, 10-year-OS rate, 64.7% vs. 78.1%, p=0.022, Figure 2B) as well as in FIGO I endometrioid cancer group (10-year-PFS rate, 70.2% vs. 81.8%, p=0.047, Figure 2C; 10-year-OS rate, 74.0% vs. 83.9%, p=0.041, Figure 2D). Neither OS nor PFS in other subgroups showed significant differences, which is most likely related to the limited number of cases and a low number of events. In order to evaluate whether EP3 immunostaining is an independent prognostic factor, multivariate analyses were conducted. The biomarker and clinicopathological variables which have a great impact on the regression coefficient of EP3 were involved in the analysis, including age, stage, grading, and recurrence. After adjusting for these factors, the expression of EP3 in the overall cohort was showed to be an independent prognostic marker for PFS (HR 1.014, 95%CI 1.003-1.024, p = 0.01) (Table 2) but not for OS (HR 1.008, 95%CI 0.998-1.019, p = 0.122). C-index for PFS was 0.855, indicating that the Cox model was capable of predicting the prognosis accurately [20, 21].

**Table 2:** Multivariate Cox regression analysis of all included patients regarding PFS (n=140)

Variable	Coefficient	HR (95%CI)	P Value
Age, y	0.038	1.039 (1.003-1.076)	**0.036**
FIGO stage (I vs. ≥ II)	1.038	2.824 (1.261-6.324)	**0.012**
WHO grading			0.426
1 vs. 2	-0.284	0.753 (0.309-1.835)	0.533
1 vs. 3	-0.604	0.546 (0.221-1.353)	0.192
EP3	0.013	1.014 (1.003-1.024)	**0.010**
Recurrence	2.957	19.240 (7.710-48.013)	**<0.001**

Significant results are shown in bold; HR: Hazard Ratio; CI: confidence interval.

### Correlations with other EC-related proteins

**Figure 3:**
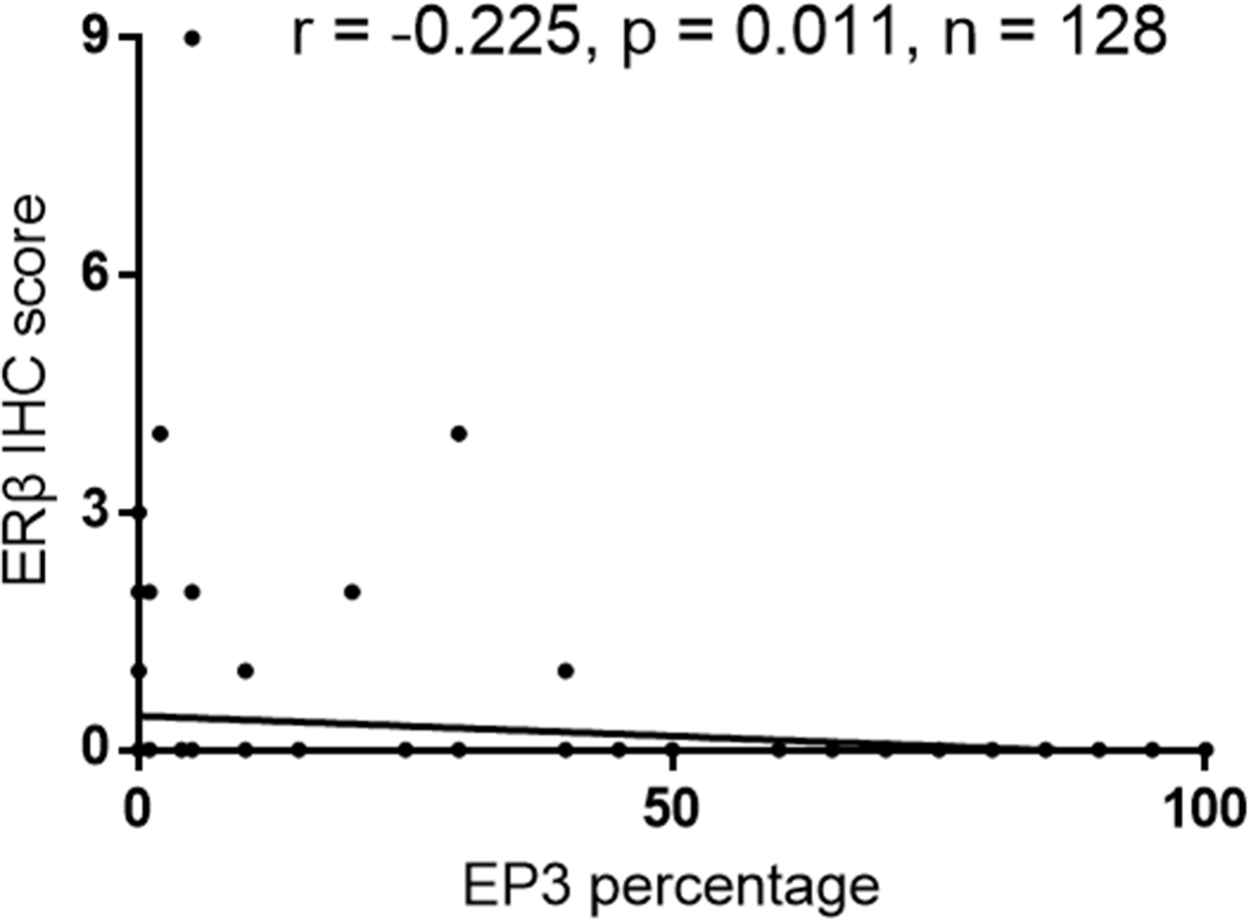
Correlation analysis of EP3 and ERβ in EC tissue (n=128)

We performed a correlation analysis to evaluate the association of EP3 with proteins that are related to EC. The staining of EP3 showed no statistically significant correlation with ERα (correlation coefficient r = -0.15; p = 0.091), progesterone receptor A (PRA) (correlation coefficient r = -0.095; p = 0.286), PRB (correlation coefficient r = -0.023; p = 0.793), and glycodelin A (GdA) (correlation coefficient r = 0.153; p = 0.88). However, we found a significant negative correlation between EP3 and ERβ (correlation coefficient r = -0.225; p = 0.011) (Figure 3).

### PGE2 enhances EP3 expression in EC cells

**Figure 4:**
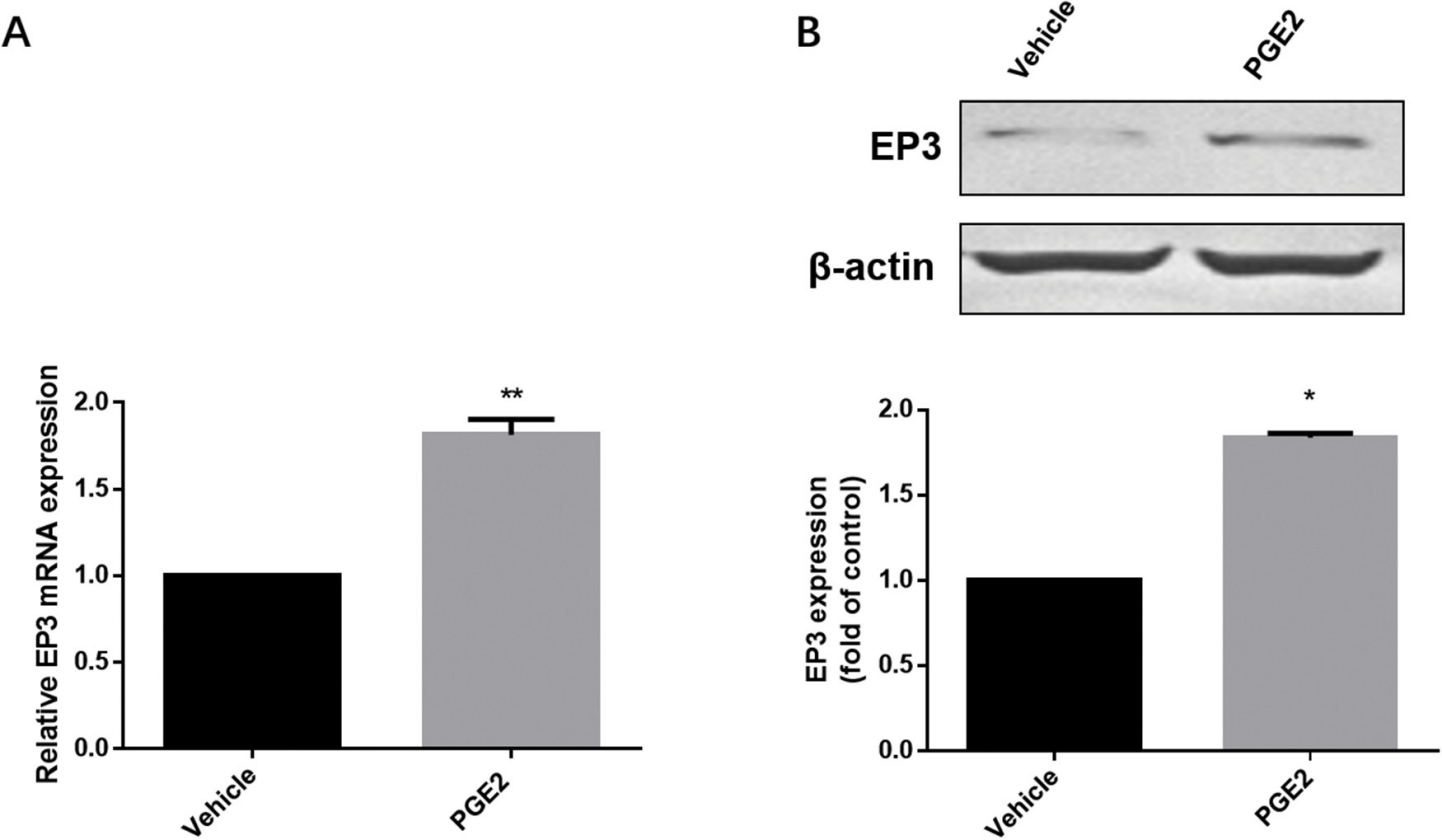
The effect of PGE2 on EP3 in RL-952 cells **(A)** Cells treated either with vehicle (0.1% (v/v) DMSO) or 1μM PGE2 for 4h and subjected to RT-PCR. Bar graph represents mean ± SEM (n = 6). ^**^p < 0.001 (compared to vehicle treated control group). **(B)** Cells treated either by vehicle (0.1% (v/v) DMSO) or 1μM PGE2 for 12h and subjected to western blotting. Histogram represents the ratio of EP3 to β-actin as assessed with pooled densitometric data. Data was normalized to the expression of vehicle treated group. β-actin was used as loading control. Bar graph represents mean ± SEM (n = 3). ^*^p < 0.01 (compared to vehicle treated control group). For gel source data, see Supplementary Figure 4.

Available EC cell lines were analyzed for EP3 expression, identifying RL95-2 and HEC-1-A with high and moderate levels of EP3 protein, respectively, compared to Ishikawa and HEC-1-B with low and undetectable EP3 expression levels (Supplementary Figure 2). According to these results, we chose RL95-2 cell line as the model for our functional EP3 study. To confirm the effect of PGE2 on EP3, we examined both mRNA and protein levels of EP3 in RL95-2 cells after exposure to PGE2. Both EP3 mRNA and protein were noted to be increased following PGE2 treatment (Figure 4).

### Proliferation of EC cells is inhibited by EP3 antagonist

**Figure 5:**
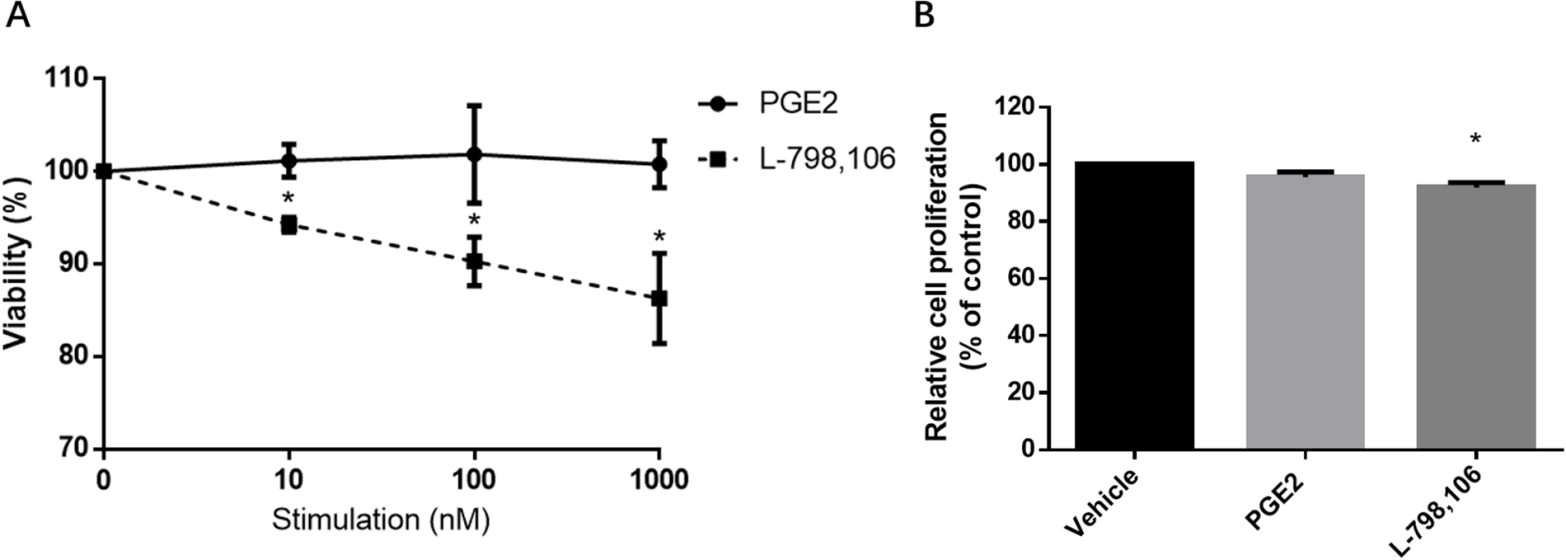
EP3 antagonist but not PGE2 suppresses proliferation of RL95-2 cells **(A)** Cells cultured with indicated concentrations of PGE2 or L-798,106 for 48 h. The viability was determined by MTT assay. Results are normalized to cell viability of control group (0.1% (v/v) DMSO). Bar graphs represent mean ± SEM (n = 3). ^*^p < 0.05 (compared to vehicle treated control group). **(B)** Cells treated with vehicle (0.1% (v/v) DMSO), 1μM PGE2 or 1μM L-798,106 for 48 h. Proliferation was determined by BrdU assay. Results were normalized to cell proliferation of control group. Bar graphs represent mean ± SEM (n = 6). ^*^p < 0.01 (compared to vehicle treated control group).

After 48 hours of treatment with 10, 100, 1000 nM PGE2, the EP3 antagonist L-798,106 or the vehicle control (DMSO, 0.1%), MTT assay was used to assess viability. L-798,106 significantly decreased viability in a dose-dependent manner compared to control group, consistent with a pro-proliferative effect of EP3 (Figure 5A). Given that MTT assay is designed to measure the number of metabolically active cells, we conducted a BrdU assay, which assesses the proliferative cells by quantifying the BrdU incorporated into DNA during the S-phase [22]. Aligned with MTT, BrdU also indicated that EP3 antagonist inhibited the cells proliferation (Figure 5B). Contrarily, neither MTT nor BrdU showed a changed proliferation of RL95-2 following PGE2 exposure (Figure 5).

### Migration of EC cells is inhibited by EP3 antagonist

**Figure 6:**
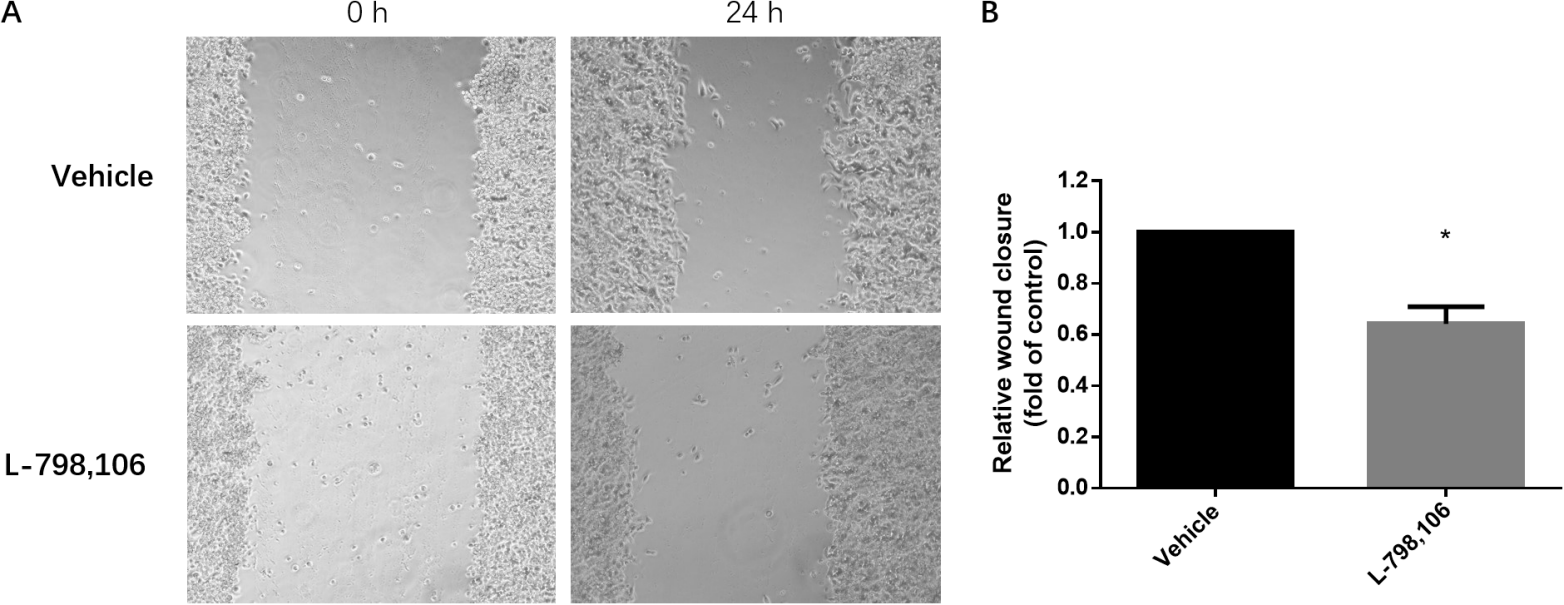
EP3 antagonist inhibits migration ability of RL95-2 cells **(A)** Representative images show cell migration into the wounded area in vehicle treated group and 1 μM L-798,106 treated group. **(B)** Histogram compares migration in vehicle treated group and 1 μM L-798,106 treated group. Results were normalized to cell proliferation of control group. Bar graph represents mean ± SEM (n = 4). ^*^p < 0.05 (compared to vehicle treated control group).

To identify whether EP3 could facilitate metastasis of endometrial cells, we performed wound healing (scratch) assay. The results showed that after treatment of EP3 antagonist, the migration ability of RL95-2 cells was significantly suppressed compared to that in the vehicle group (Figure 6).

### Inhibited EP3 increases ERβ expression and decreases Ras activity in EC cells

**Figure 7:**
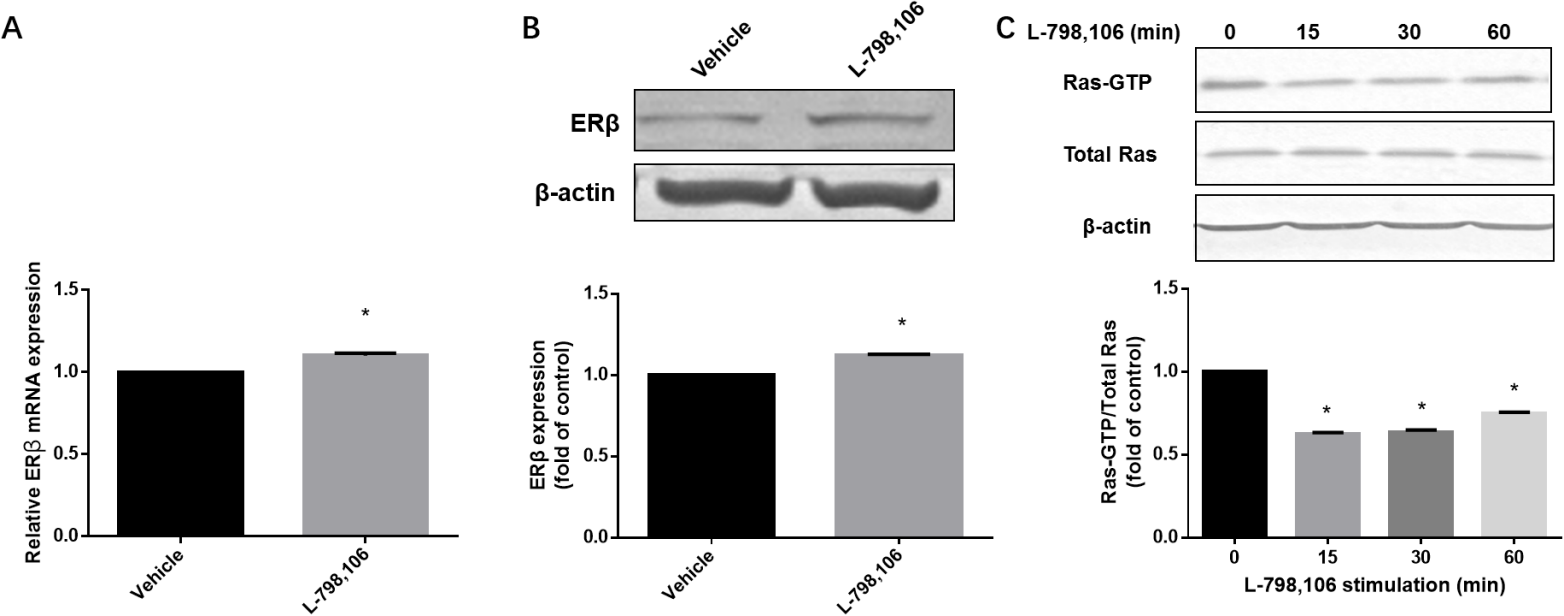
EP3 antagonist increases expression of ERβ and decreases activity of Ras in RL95-2 cells **(A)** Cells treated either by vehicle (0.1% (v/v) DMSO) or 1μM L-798,106 for 4 h and subjected to RT-PCR. Bar graph represents mean ± SEM (n = 4). ^*^p < 0.001 (compared to vehicle treated group). **(B)** Cells treated either by vehicle (0.1% (v/v) DMSO) or 1μM L-798,106 for 12 h and subjected to western blotting. Histogram illustrates the ratio of ERβ to β-actin as assessed with pooled densitometric data. Data was normalized to the expression of vehicle treated group. β-actin was used as loading control. Bar graph represents mean ± SEM (n = 3). ^*^p < 0.001 (compared to vehicle treated group). **(C)** Cells incubated with 1μM L-798,106 for indicated time and subjected to western blotting. Data was normalized to the expression at 0 min. β-actin was used as loading control. Bar graph represents mean ± SEM (n = 3). ^*^p < 0.001 (compared to 0 min group). For gel source data, see Supplementary Figure 4.

As immunohistochemistry showed a negative association of EP3 with ERβ, we further investigated whether EP3 acts upstream of ERβ. Both ERβ mRNA and protein were upregulated by L-798,106 treatment (Figure 7A, 7B). A previous study demonstrating that ERβ acts as a inhibitory signaling molecule upstream of Ras, prompted us to examine the activity of Ras in response to L-798,106 treatment [23]. As shown in Figure 7C, EP3 antagonist significantly decreased the activity of Ras in a time-dependent manner.

### EP3 does not change the estradiol biosynthesis

Since EP3 can change the expression of ERβ, we speculated it might also have some effect on estradiol biosynthesis. According to a previous report, EC cell lines can form estradiol from estrone [24]. To verify our hypothesis, we estimated the estradiol concentration of EC cells exposed to L-798,106 together with 10% FBS, which contains estrone. Given that the conversion can be observed after 24 h incubation [24], we set the incubation time point as 24 h. However, EP3 antagonist did not affect the formation of estradiol (Supplementary Figure 3).

## DISCUSSION

In this study of 140 EC tumor samples, we confirmed that EP3 is expressed in EC tissues and showed for the first time that EP3 expression in glandular epithelial cells correlates with tumor grade and is associated with impaired prognosis regarding PFS and OS. For EC, the results of our explorative analysis could show that EP3 might serve as a novel diagnostic and therapeutic target strongly deserving further investigation.

Furthermore, we demonstrated that EP3 might also play a role in regulating endometrial tumor growth. *In vitro*, we could show that EP3 antagonist may attenuate proliferation and migration of EC cells, which supports the possible significance of EP3 in oncogenesis as previously reported. EP3 knockout mice have shown to markedly reduce tumor growth and tumor-associated angiogenesis [25]. By activating downstream signaling pathways, such as protein kinase A (PKA) [16], extracellular signal-regulated kinases 1 and 2 (ERK1/2) [26] and phosphoinositide 3-kinase (PI3K)/Protein Kinase B (AKT)/Glycogen synthase kinase 3 beta (GSK-3β) pathway [27], EP3 enhances the viability [16], proliferation [28], and invasiveness [15] of various cancer cells. Besides the direct influence on cancer cells, EP3 also promotes the tumor metastasis and angiogenesis by upregulating the matrix metalloproteinases (MMP)-9 of endothelial cells [29], which is an essential component of stroma and constitutes the tumor microenvironment. All these studies support our finding regarding the anti-cancer function of EP3 antagonist in EC cells.

In contrast to a previous report [12], our study did not confirm an effect of PGE2 on EC cells, even though PGE2 was proved to upregulate the expression of EP3 in our study. This might underline the fact that aspirin instead of non-aspirin NSAIDs reduces the risk of EC [9]. Aspirin has been reported to regulate the growth of prostate cancer cells in a PGE2-independent way [14]. There might also be a PGE2-independent pathway in EC cells. Alternatively, the nonspecific binding of PGE2 to other receptors, such as EP2 and EP4, which have been known to increase cyclic adenosine monophosphate (cAMP) via Gs protein [13], might counteract the effect of EP3. This is the main reason we chose L-798,106, a highly selective antagonist of EP3 for our study to avoid interference caused by nonspecific binding. The same study showed EP3 did not influence proliferation of Ishikawa cell line [12]. These discrepancies about EP3 and PGE2 might be due to the usage of different cell lines as an experimental model. The Ishikawa cell line, used by the other group, barely expresses EP3 according to our data and thus could be less sensitive to EP3 pharmacological stimulation.

For the first time, we could show that EP3 was negatively associated with ERβ in EC tissue. Pharmacological research provides more information to verify that EP3 regulates ERβ, although the mechanism is far from clear. L-798,106 increased ERβ expression in RL95-2 cells. ERβ belongs to nuclear receptor superfamily and is clarified to be a modulator of ERα by functioning oppositely [30] and by repressing ERα transcriptional activity [31]. Although the effect of ERβ in EC has not been fully elucidated, accumulating evidence reveal that ERβ has a protective effect on the endometrium [32–34] and promotes differentiation and inhibits proliferation as well as invasion of endometrium [30, 35, 36]. In addition, ERβ is also shown to inhibit the migration of cancer cells [37].

Ras, a GTPase, transduces different downstream signalings by interacting with various Ras-effectors, such as Raf kinase, PI3K, and Ral guanine nucleotide exchange factors (Ral-GEFs) [38] and plays an important role in multistep carcinogenesis in EC [39, 40]. The activation of Ras can be induced by either mutant Ras or alteration of Ras protein expression. Here, we confirmed that EP3 antagonist decreased active Ras. Since ERβ can modulate Ras signaling [23], EP3 might regulate phenotypes of RL95-2 cells via ERβ/Ras.

As EP3 does not affect estradiol biosynthesis, the regulation of ERβ apparently was not induced by estradiol. The main effect of EP3 is to bind to the Gi protein and inhibit cAMP production [41], which is documented to increase the ERβ protein expression rather than ERα protein expression and promote ERβ transcriptional activation [42]. Therefore, EP3 might negatively regulate ERβ by adjusting the cAMP concentration.

Also, an inhibitory effect of EP3 on tumor development has been observed. In prostate and colon cancer, EP3 mRNA was remarkably reduced compared to corresponding normal tissue and EP3 impaired growth ability of these two cancer cell lines [14, 17]. Furthermore, EP3 was reported to inhibit the hormone-dependent growth of breast cancer by reducing aromatase activity of adipose stromal cells [43]. Among all the prostaglandin receptors, EP3 is the most complicated receptor because of its various isoforms. The human EP3 gene, consisting of ten exons and generating nine mRNAs, encodes at least eight distinct EP3 splice variants, which only differ at C-terminal tails [44]. The specific C-terminal tails produced by alternative splicing bind to different G proteins and activate different second messengers, which consequently determines the diverse physiological activity of EP3 receptor [19, 28, 41, 44–48]. This might partly explain these cell and tissue type specific phenomena. Further research on the specific isoforms expressed in EC and their effects should be performed.

Over the past years, molecular cancer biology has been integrated into the clinical routine of different tumor entities (e.g., breast cancer [49]). In this context, the Cancer Genome Atlas Research Network proposed a new classification of EC and categorized EC as POLE ultramutated, microsatellite instability hypermutated, copy-number low, and copy-number high [50]. The four TGCA subtypes are related to different clinical outcomes, among which the copy-number high subgroup has impaired PFS, while the POLE ultramutated subgroup has improved PFS [50]. Mutations of RPL22 are almost exclusively identified in the microsatellite instability (MSI) group [50]. Direct sequencing of RPL22 exons 2 and 4 in 226 MSI endometrial tumors confirmed 51.6% tumors were heterozygous for the 43delA mutation, which was also presented in RL95-2 cell line [51]. Consequently, we speculate that this cell line could represent the MSI group, the survival of which is centered. Although not yet fully implemented in clinical routine, this classification gains more prognostic significance so that our results on EP3 will need to be confirmed in this context in future investigations.

In conclusion, we demonstrate for the first time that EP3 expression in glandular epithelial cells is associated with advanced WHO grading and poor patients’ prognosis. Inhibited EP3 mediates an anti-cancer effect in EC cells, which can be utilized for therapeutic interventions. As the results are partially contradictory to previous studies in other cell lines and tumor entities, our study indicates that EP3 seems to act in a cell and tissue type-specific manner. For EC, we could show that EP3 might serve as a novel diagnostic and therapeutic target strongly deserving further investigation.

## MATERIALS AND METHODS

### Patients

Formalin-fixed paraffin-embedded tissue of 140 patients, who received surgery for EC at the Department of Obstetrics and Gynecology of the Ludwig-Maximilians-University Munich between 1990 and 2002 was available. All patients provided informed consent before surgery. Staging and grading were assessed by two gynecological pathologists according to the criteria of FIGO and WHO. Follow-up data were obtained from the Munich Cancer Registry. PFS was defined as the time from operation to relapse or death from any cause, whereas OS was the time from diagnosis to the death or date of the last follow-up.

The study was performed according to the Declaration of Helsinki 1975. We used the remaining material of the tumor tissue after the initial histopathological diagnosis had been completed. The current study was approved by the Ethics Committee of the Ludwig Maximilians University, Munich, Germany (approval number 063-13). Authors were blinded for clinical information during experimental analysis.

### Immunohistochemistry

Immunohistochemistry was performed as previously described by our lab [52]. Paraffin-embedded and formalin-fixed EC samples were incubation with the polyclonal rabbit IgG anti-EP3 antibody (Abcam), which was diluted at the ratio of 1:300, overnight. The signal was amplified with HRP-polymer (Zytochem-Plus HRP Polymer-kit, Zytomed, Berlin, Germany) for 30 min followed by incubating with diaminobenzidine (Dako, Hamburg, Germany) for 2.5 min. In the end, counterstaining with hematoxylin was carried out.

To support the validity of the EP3 staining, we used slides made from one normal colon tissue as the positive and negative control (Supplementary Figure 1). The negative control was performed by substituting for the primary antibody with a pre-immune serum (Rabbit Super Sensitive™ Negative Control, Hague, the Netherland).

The immunostaining of EP3 showed dots and an uneven distribution. The intensity of staining varied considerably within one slide. Therefore, the scoring was made according to the percentage of immunostained glands. The estimation of the percentage of EP3-positive glands was conducted by viewing the tumor area at 5x and 10x objectives and the results were recorded as an exact percentage. All slides were evaluated by two independent investigators. The staining and scoring with primary antibody ERα, ERβ, PRA, PRB, and GdA were performed as previously described by our research team [53, 54].

### Cell culture and drugs

One well-differentiated cell line, Ishikawa and three moderate-differentiated cell lines, RL95-2, HEC-1-A, and HEC-1-B cells were purchased from European Collection of Cell Culture (ECACC, Salisbury, UK) and maintained in RMPI 1640 medium containing 10% fetal bovine serum (FBS, Thermo Fisher Scientific) without antibiotic at 37°C in a humidified 5% CO_2_ incubator. In each experiment, cells were seeded in wells overnight before being incubated with test substances or dimethyl sulfoxide (DMSO) unless otherwise indicated. PGE2, L-798,106, a highly selective EP3 antagonist, and EP3 agonist, sulprostone was purchased from Tocris Bioscience. The results of MTT and BrdU showed that the agonist we chose did not affect proliferation of EC cells. Although Abrahao et al. [55] and Fujino et al. [26] claimed sulprostone to be a selective EP3 agonist, it has been shown that sulprostone is an EP1/EP3 dual agonist [13, 48]. As other EP3 highly selective agonists such as ONA-AE-248 are currently not commercially available, we decided to concentrate on the antagonist L-798,106 to avoid interfering effects caused by non-specific binding.

### RNA isolation and reverse transcription

Total RNA was obtained from cultured cells using a RNeasy Mini Kit (Qiagen, Hilden, Germany) and converted to cDNA with an MMLV Reverse Transcriptase First-Strand cDNA synthesis kit (epicentre, madison, WI, USA) as instructed by the manufacturer.

### Quantitative real-time RT-PCR

20μL reaction mixture containing 1μL TaqMan^®^ Gene Expression Assay 20 x (Applied Biosystems, target PTGER3, Nr. Hs00168755_m1, target ESR2, Nr. Hs01100353_m1, target ACTB, Nr. Hs99999903_m1), 10μL TaqMan^®^ Fast Universal PCR Master Mix 2 x (Applied Biosystems), 1μL cDNA template and 8μL RNase-free water was prepared per probe on an Optical Fast 96-well plate (Applied Biosystems) and covered by an optical adhesive film. PCR assays were run by using Applied Biosystems 7500 Fast Real-time PCR system. Enzymes activation was performed at 95°C for 20 s on hold. Afterward, 40 cycles of qPCR denaturing at 95 °C for 3 s and annealing at 60°C for 30 s were run. The comparative CT method, also referred to as the ∆∆CT method was applied for the results. For ∆CT values calculation, β-actin was used as an endogenous control. The results are representative of at least three independent experiments.

### Western blotting and Ras-GTP assay

The procedure and protocol of western blotting were previously described by our group [56]. Briefly, cell lysates were electrophoresed by SDS-PAGE and transferred to PVDF membranes. The membranes were incubated overnight with 1:2000 dilution of EP3 antibody (ab117998, Abcam), 1:200 dilution of ERβ antibody (53472, Anaspec) or 1:1000 dilution of β-actin antibody (A5441, Sigma). Afterwards, membranes were washed and incubated for 1 h with 1:1000 dilution of the corresponding alkaline phosphatase-conjugated secondary antibodies. Blotting was detected and visualized by BCIP/NBT Color Development Substrate (Promega). GTP-bound RAS was determined using the active Ras detection kit (8821, Cell Signaling) according to the manufacturer’s instruction.

Images were analyzed by an image analyzer (Molecular Imager^®^ Gel DocTM XR+, Bio-rad) using software Quantity One 4.6.7 (Bio-Rad, Munich, Germany). β-actin was used as an endogenous control (Supplementary Figure 4).

### Cell viability assay

RL95-2 cells were seeded at the density of 1,5 × 10^4^ cells/well in 96-well plates in sextuplicate. The next morning cells were incubated with different concentration of PGE2 or L-798,106 for 48 h. 5 mg/mL MTT [3-(4,5-dimethhylthiaoly)-2,5-diphenyltetrazolium bromide] (Sigma) in phosphate-buffered saline (PBS) was prepared. 20 μg MTT solution was added to each well for 1.5 h at 37°C. The culture medium along with MTT was then removed. 200 μL DMSO was added to each well to dissolve the visible formazan crystals, followed by mixed thoroughly on the shaker for 5 min at room temperature. The optical density (OD) was read at 595 nm using Elx800 universal Microplate Reader.

### Cell proliferation assay

RL95-2 Cells were seeded in 96-well plates at 5 × 10^3^ cells/well in sextuplicate. The next morning cells were incubated with different concentration of PGE2 or sulprostone for 48 h. 5-Bromo-2'-Deoxyuridine (BrdU) incorporation assay (11647229001, Roche) was used to determine the cell proliferation according to manufacturer’s protocols. OD was quantified at 450 nm using Elx800 universal Microplate Reader.

### Wound healing (scratch) assay

1.4 × 10^6^/well RL95-2 cells were cultured in 24-well-plates overnight. The next morning the central fields of confluent monolayers were scratched with a 200 μl pipette tip to make artificial wound gaps. The detached cells were aspirated and rinsed once with PBS. 1 μM L-798,106 was added to treat cells. After 0 h, 24 h, cell migration was monitored by photographing using an inverse phase contrast microscope (Leica Dmi1, Leica, Wetzlar, Germany) with a camera (LEICA MC120 HD, Leica, Wetzlar, Germany). Photomicrographs of wounded areas covered by cells were analyzed by software Image J (http://rsb.info.nil.goc/ij). The cell migration area = area at 0 h – area at 24 h.

### Estradiol measurements

3× 10^5^/well RL95-2 cells were seeded in 24-well-plates. After treated by 1 μM L-798,106 for 24 hours, the supernatant was collected and centrifuged (13,200 g, 10 min) to remove cell debris. The estradiol concentration was subsequently determined using chemiluminescent immunometric assay (IMMULITE 2000 immunoassay system) (Siemens, Germany) as described by the manufacturer.

### Statistical analysis

A Student’s t-test (two-tailed) was used to analyze means of two groups. Mann-Whitney U or Kruskal-Wallis tests were conducted to compare non-parametric variables between or among groups. A Spearman rank test was performed for correlations between continuous variables. Survival times were compared using Kaplan-Meier (log-rank) test method. The ROC curve was drawn to identify an appropriate cut-off. The ROC curve analysis is one of the most widespread methods used in cut-off point selection. The ROC curve is a plot, y-axis of which represents sensitivity and x-axis of which represents (1-specificity) [57]. Youdan index, defined as the maximum (sensitivity+specificity-1) [58], is applied to ensure the optimal cut-off which can maximize the sum of sensitivity and specificity [59, 60]. A cox-regression model for multivariate analyses was used. Harrell’s c-index was performed to evaluate the accuracy of the Cox model using R 3.3.1 with Hmisc and rms packages (http://www.r-project.org). A p-value below 0.05 was considered statistically significant for all analyses. The data were analyzed using the Statistical Product for Social Science (SPSS, IBM, Armonk, NY, USA) version 23.0.

## SUPPLEMENTARY MATERIALS FIGURES


