# Supplementary material for: Prostaglandin receptor EP3 regulates cell proliferation and migration with impact on survival of endometrial cancer patients
Source: Oncotarget. 2017 Dec 9;9(1):982–94. doi: 10.18632/oncotarget.23140 (PMC5787529; doi:10.18632/oncotarget.23140)
Supplement: Supplementary file 1 [file oncotarget-09-982-s001.pdf]

Prostaglandin receptor EP3 regulates cell proliferation and migration with impact on survival of endometrial cancer patients

SUPPLEMENTARY MATERIALS

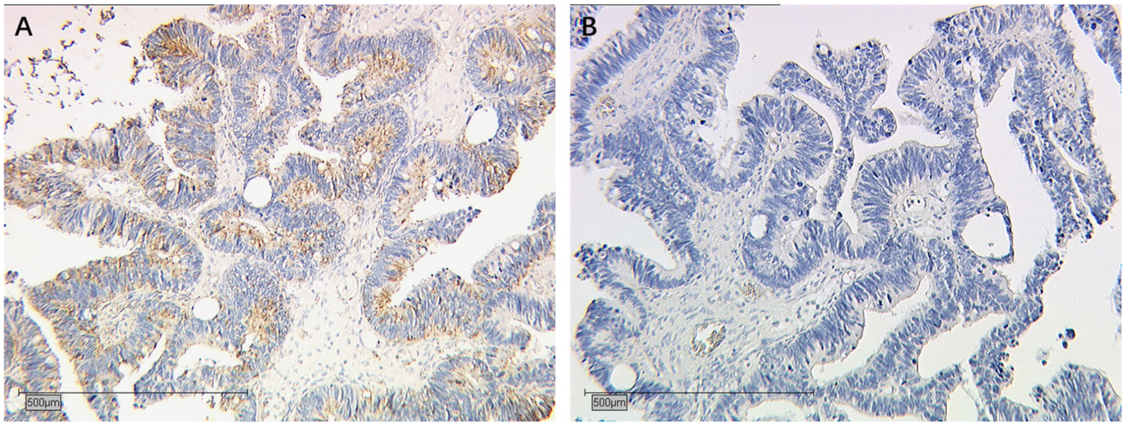

Supplementary Figure 1: Representative microphotographs of positive and negative controls for EP3 in normal colon tissue. (A) Positive control of EP3 staining in normal colon tissue showed strong cytoplasm expression in more than 80% epithelial cells. **(B)** Negative control of EP3 staining in same normal colon tissue as positive control. Scale bars equal 500 µm.

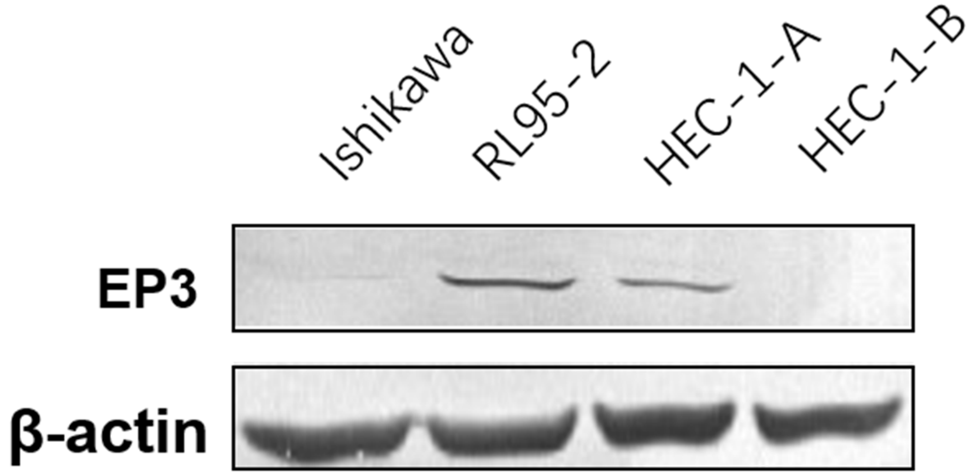

Supplementary Figure 2: EP3 protein expression in various EC cells. For gel source data, see Supplementary Figure 4.

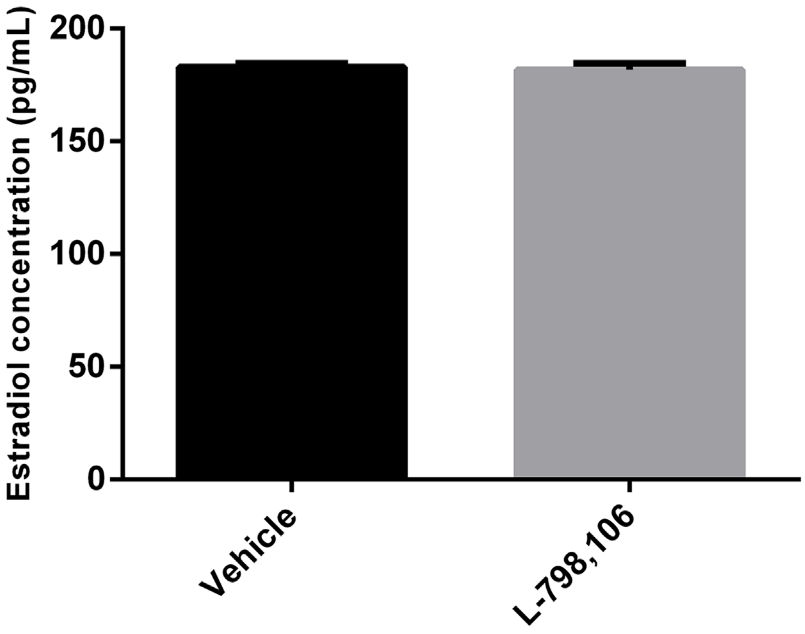

Supplementary Figure 3: EP3 does not change the formation of estradiol. RL95-2 cells incubated with vehicle (0.1% (v/v) DMSO) or 1 µM L-798,106 for 24 h. Levels of estradiol in the supernatant were estimated by chemi-luminescent immunometric assay. Bar graph represents mean ± SEM (n = 4).

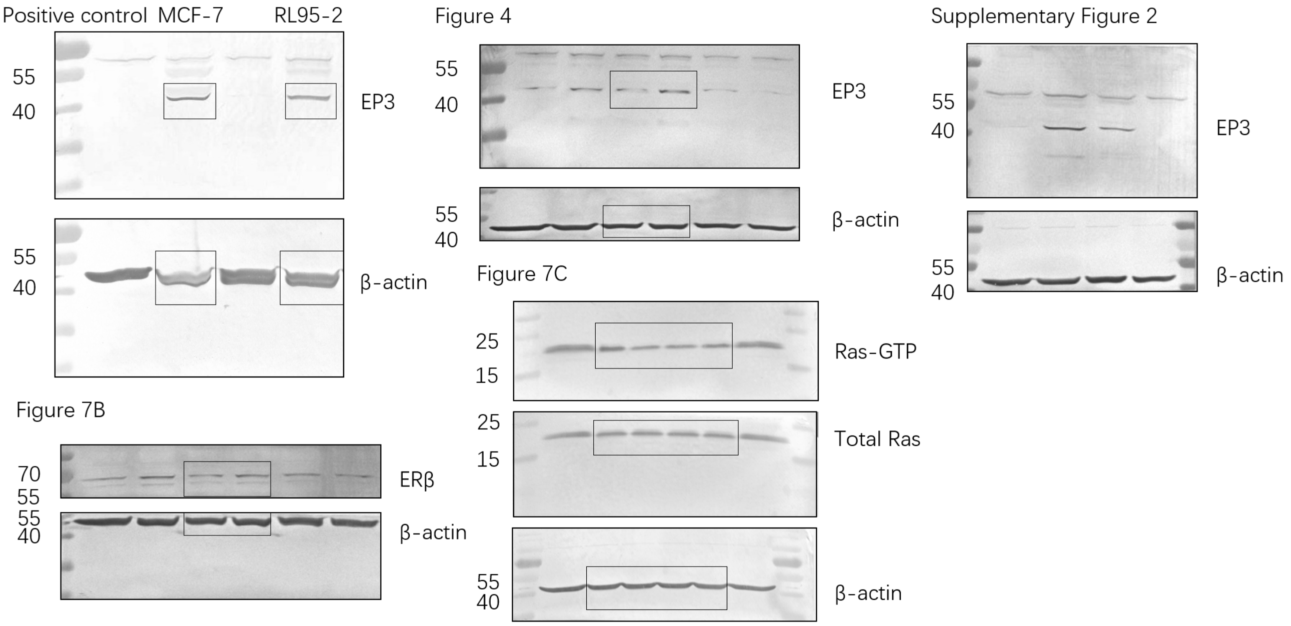

Supplementary Figure 4: Uncropped scans with marker indications.
